# The Significance of Thiol/Disulfide Homeostasis and Ischemia-modified Albumin Levels in Assessing Oxidative Stress in Obese Children and Adolescents

**DOI:** 10.4274/jcrpe.galenos.2019.2019.0039

**Published:** 2020-03-19

**Authors:** Eda Mengen, Seyit Ahmet Uçaktürk, Pınar Kocaay, Özlem Kaymaz, Salim Neşelioğlu, Özcan Erel

**Affiliations:** 1 Ankara City Hospital, Children’s Hospital, Clinic of Pediatric Endocrinology, Ankara, Turkey; 2 Ankara University Faculty of Medicine, Depatment of Statistics, Ankara, Turkey; 3 Yıldırım Beyazıt University Faculty of Medicine, Department of Biochemistry, Ankara, Turkey

**Keywords:** Obesity, children and adolescents, thiol/disulfide homeostasis, ischemia-modified albumin

## Abstract

**Objective:**

There is an association between obesity and several inflammatory and oxidative markers in children. In this study, we analyzed thiol/disulfide homeostasis and serum ischemia-modified albumin (IMA) levels for the first time in order to clarify and determine the oxidant/antioxidant balance in metabolically healthy and unhealthy children.

**Methods:**

This study included obese children and healthy volunteers between 4-18 years of age. The obese patients were divided into two groups: metabolically healthy obese (MHO) and metabolically unhealthy obese (MUO). Biochemical parameters including thiol/disulfide homeostasis, and IMA concentrations were analyzed.

**Results:**

There were 301 recruits of whom 168 (55.8%) were females. The obese children numbered 196 (MHO n=58 and MUO n=138) and healthy controls numbered 105. No statistically significant difference could be found in ages and genders of the patients among all groups (p>0.05, for all). Native thiol (SH), total thiol (SH+SS), and native thiol/total thiol (SH/SH+SS) ratio were statistically significantly lower in the MUO group than the control group (p<0.001, p=0.005, and p=0.005; respectively). Disulfide (SS), disulfide/native thiol (SS/SH), disulfide/total thiol (SS/SH+SS) and IMA levels were statistically significantly higher in the MUO group than the control group (p=0.002, p<0.001, p<0.001, and p=0.001, respectively).

**Conclusion:**

Chronic inflammation due to oxidative stress induced by impaired metabolic parameters in MUO children caused impairment in thiol redox homeostasis. Our data suggested that the degree of oxidant imbalance in obese children worsened as obesity and metabolic abnormalities increased. It is hypothesized that thiol/disulfide homeostasis and high serum IMA levels may be reliable indicators of oxidant-antioxidant status in MUO children.

**What is already known on this topic?**

Although different mechanisms are proposed for the pathogenesis of complications associated with obesity, the most widely accepted hypothesis is that adipose tissue inflammation plays a critical role.

**What this study adds?**

Chronic inflammation due to oxidative stress induced by impaired metabolic parameters in metabolically unhealthy obese (MUO) children caused impairment in thiol redox homeostasis. Our data suggested that the degree of oxidant imbalance in obese children worsened as obesity and metabolic abnormalities increased. It is hypothesized that thiol/disulfide homeostasis and high serum ischemia-modified albumin levels may be reliable indicators of oxidant-antioxidant status in MUO children.

## Introduction

Childhood obesity is one of the most important health problems of the 21^st^ century (1). This is a global problem and continuously affects urban populations of many low and middle-income families and the prevalence is increasing at an alarming rate. A recent study proposed that 1.48 billion adults in the world are overweight, 502 million adults are obese, and 180 million children are overweight or obese (2). There has been a dramatic increase in the prevalence of overweight and obesity in the adult Turkish population, reaching figures as high as 30-40% (3).

Although different mechanisms are proposed for the pathogenesis of complications associated with obesity, the most widely accepted hypothesis is that adipose tissue inflammation plays a critical role, and oxidative stress (OS) appears in obese individuals (4). OS is the loss of the normal homeostatic balance between reactive oxygen species (ROS) and antioxidant substances. OS is toxic to cells by causing membrane lipid peroxidation and membrane damage (5). Thiols are important antioxidants and play a role in non-enzymatic elimination of ROS. Thiol/disulfide homeostasis is necessary for some detoxification mechanisms. Previous studies have reported parameters of thiol/disulfide homeostasis which include native thiol, total thiol, and disulfide concentrations and disulfide/native thiol, native thiol/total thiol, and disulfide/total thiol ratios (6,7,8,9,10,11,12,13). Dynamic thiol/disulfide homeostasis plays a key role in antioxidant protection, detoxification, signal transduction, apoptosis, regulation of enzymatic activity, the function of some transcription factors and some cellular signalling mechanisms (14,15). Moreover, dynamic thiol/disulfide homeostasis has been implicated in the pathogenesis of many disorders (16,17,18,19,20,21,22,23,24,25).

Ischemia-modified albumin (IMA) is produced through the modification of albumin by ROS produced as a result of ischemic episodes (26). High IMA concentrations have been used to predict cardiovascular risk in obese children and to evaluate subclinical vascular disease in patients with diabetes mellitus (26,27).

The aim of this study was to evaluate antioxidant status in obese children with a focus on both markers of thiol/disulfide homeostasis and IMA. This is the first study to measure both thiol/disulfide homeostasis and serum IMA concentrations in metabolically healthy obese (MHO) and metabolically unhealthy obese (MUO) children in order to clarify and determine their roles in oxidant/antioxidant balance. In addition, the effect of obesity in metabolically unhealthy children on biomarkers of OS was investigated.

## Methods

### Study Design and Patient Selection

This case-control study was conducted in Ankara Pediatric Hematology and Oncology Training and Research Hospital between May-2018 and July 2018 and included obese children and healthy controls aged between 4-18 years. Exclusion criteria for obese patients were the presence of any hepatic, renal, cardiac, autoimmune, infectious, musculoskeletal or malignant diseases, taking any vitamin supplementation, or drug use that might lead to obesity and the presence of any chromosomal, endocrine or genetic syndromes. The control group included 105 healthy children without any known chronic or acute disease. The control group consisted of sex- and age-matched healthy subjects who were of normal weight for age. In addition, none of the control group had insulin resistance (IR), impaired fasting glucose, dyslipidemia or hypertension.

Obese patients were divided into two groups. Subjects who did not have dyslipidemia, impaired fasting glucose, IR, hepatosteatosis or hypertension were accepted as MHO and those who had at least one of these conditions were accepted as MUO, as previously described (28). Clinical and laboratory findings of the obese and control groups were compared.

Weight measurements were performed with the subjects dressed in thin clothes and without shoes, using an electronic weighing device with the SECA 274 Stadiometer (Hamburg, Germany) with 1 mm accuracy. Height measurements were performed with the Ayrton^®^ Stadiometer (5322 Frost Point Prior Lake, MN 55372, USA), sensitive to a 0.1 cm difference in an upright position with bare feet. Body mass index (BMI) was calculated with the standard formula: [weight (kg) / height^2 ^(m^2^)]. For statistical evaluation, BMI-standard deviation score (BMI-SDS) was used. Patients whose BMI-SDS were >2 were accepted as obese (29). The BMI-SDS values were calculated using the reference values developed by Neyzi et al (30).

Routine physical examinations were performed in all obese subjects and controls. Puberty was assessed in all obese subjects and controls by Tanner staging. In girls, stage 2 breast development and in boys 4 mL testis volume were accepted as indicating the start of puberty (31,32).

Blood pressure measurement was performed from the right arm in the sitting position after 15 minutes of resting, using a mercury sphygmomanometer (ERKA, Germany). If the blood pressure was above the 95^th^ percentile according to age, gender and height, two more measurements were obtained. Hypertension was accepted if two of the three measurements were at or above the 95^th^ percentile (33).

### Laboratory Analysis

Blood samples were obtained after 8-10 hours of fasting. Fasting plasma glucose (FPG), fasting plasma insulin, alanine aminotransferase, aspartate aminotransferase, total cholesterol (TC), triglyceride (TG), and high-density lipoprotein cholesterol (HDL-C) concentrations were measured. Low-density lipoprotein cholesterol (LDL-C) concentrations were measured using the Friedewald formula (34). Serum glucose and lipid profile measurements were performed using the Roche modular system/Integra 800 device and kit (Mannheim, Germany). FPG between 100-125 mg/dL was accepted as “impaired fasting glucose” (35). A TC concentration of ≥200 mg/dL, TG ≥150 mg/dL, HDL-C ≤35 mg/dL and LDL-C ≥100 mg/dL was accepted as dyslipidemia (36).

Fasting insulin, thyroid-stimulating hormone (TSH) and free T4 (fT4) concentrations were measured using enzymatic immunoassay method using a Beckman Coulter DXI 800 device (Brea, CA, USA). Reference values for Beckman Coulter TSH and fT4 kits used in our hospital were 0.7-5.69 µIU/mL for TSH and 0.65-1.06 ng/dL for fT4. IR was calculated using the Homeostasis Model Assessment of fasting IR (HOMA-IR) method with the following formula: FPG (mmol/L) x fasting insulin (mIU/mL)/22.5 (37). The HOMA-IR cut-off value for prepubertal subjects was taken as 2.5 and for pubertal subjects as 4 (38).

Hepatosteatosis was evaluated in our radiology clinic with upper abdominal ultrasonography using a Toshiba Xaria I Style Ultrasound device (Tokyo, Japan). Liver ultrasound findings were staged as follows: normal liver appearance (no hepatosteatosis), mild (stage 1), moderate (stage 2) and severe hepatosteatosis (stage 3) (39).

### Measurement of Serum Ischemia-modified Albumin

Blood samples were obtained using anticoagulant-free tubes and centrifuged for five minutes at 3500 rpm. Serum aliquots for measuring IMA blood concentrations were pipetted into Eppendorf tubes and stored at -80 °C until testing. Serum IMA concentrations were measured by a colorimetric method described by Bar-Or et al (40) and results were reported as absorbance units (ABSU).

### Measurement of Thiol/Disulfide Homeostasis Parameters

Blood samples were obtained between 8 a.m. and 10 a.m. after 8-10 hours fasting. The samples were then centrifuged at 1500 rpm for 10 minutes. Separated serum samples were immediately frozen and stored at -80 °C until analyzed. All thiol/disulfide parameters were studied in the same samples. Serum concentrations of native and total thiol and ratios of disulfide, and native and total thiol were determined by a spectrophotometric method using an automatic clinical chemical analyzer (Roche, Cobas 501, Mannheim, Germany) as previously described by Erel and Neselioglu (41).

### Ethical Statements

The study was approved by Ankara Children’s Hematology Oncology Training and Research Hospital’s Ethics Committee (approval number: 2018-70). The study was performed in accordance with the ethical rules based on the principles of the Helsinki Declaration. Written informed consent forms were obtained (when appropriate) from the parents and the children.

### Statistical Analysis

A sample size calculation was performed considering detection of 0.20 effect size, α=0.05 and a power of 88.0% using variance analysis (one-way ANOVA). The result of the power analysis showed that the minimum number of patients required was 303. Data obtained from this study were analyzed using Statistical Package for Social Sciences for Windows, version 23.0 (IBM Inc., Armonk, NY, USA) (42). Frequency distributions and percentages were given for categorical variables. For continuous variables, assumption of normality was tested by visual (histogram and probability plots) and analytic methods (Kolmogorov-Smirnov/Shapiro-Wilk test). Descriptive statistics were presented as mean ± standard deviation or median and interquartile ranges for continuous variables as appropriate. Equality of variances was controlled with the Levene test. One-way ANOVA was used to measure the difference among three groups if parametric test conditions were met, and the Bonferroni test among *post hoc* tests was used to make binary comparisons. Kruskal-Wallis test was used when parametric test conditions were not met. Student’s t-test was used to determine whether a difference existed between two groups when parametric test conditions were met and Mann-Whitney U tests was used when conditions were not met. Chi-square (χ2) test was used for the analysis of categorical variables. For the MUO group, cut-off values of native thiol, total thiol, disulfide, disulfide/native thiol, disulfide/total thiol, native thiol/total thiol and IMA concentration were determined by using Receiver Operating Characteristic (ROC) curve analysis. Significance level of the tests was accepted to be p<0.05.

## Results

This study included 301 children, of whom 168 (55.8%) were female. Within the group, 138 (45.85%) were MUO, 58 (19.3%) were MHO and there were 105 (34.9%) healthy volunteers. The obese group consisted of 70.4% MUO and 29.6% MHO. No statistically significant difference could be found in ages and genders of the patients among all groups (p>0.05, for all). BMI-SDS, HOMA-IR, and concentrations of glucose, insulin, TC, LDL-C and TSH were higher in the obese children than in the controls. The demographic, clinical and laboratory characteristics of the participants are displayed in Table 1.

Obese patients had lower native thiol (SH) and total thiol (SH+SS) concentrations, and a lower native thiol/total thiol (SH/SH+SS) ratio than controls (p<0.001, p=0.002 and p=0.013, respectively). In addition obese patients had increased disulfide (SS) concentrations and higher disulfide/native thiol (SS/SH) and disulfide/total thiol (SS/SH+SS) ratios than the controls (p=0.005, p<0.001 and p<0.001, respectively). Moreover, serum IMA concentration was significantly higher in the obese group (p<0.001). Thiol/disulfide homeostasis parameters and comparison of IMA concentrations between the control and the obese groups are given in Table 2.

Native thiol (SH) and total thiol (SH+SS) concentrations and native thiol/total thiol (SH/SH+SS) ratio were statistically significantly lower in the MUO group than the control group (p<0.001, p=0.005 and p=0.005, respectively). Disulfide (SS) and IMA concentrations and disulfide/native thiol (SS/SH) and disulfide/total thiol (SS/SH+SS) ratios were statistically significantly elevated in the MUO group compared with the control group (p=0.002, p<0.001, p<0.001 and p=0.001, respectively). Comparison of dynamic thiol/disulfide homeostasis parameters and IMA concentrations among the groups are given in Table 3.

All patients with a BMI-SDS >3 were categorized as a subgroup and were investigated separately for the study parameters. This subgroup included 14 patients from the MHO and 24 patients from the MUO groups. Interestingly, all parameters were similar in both groups (p>0.05, for all; Table 4).

ROC curve analysis was performed for the MUO group for native thiol, total thiol, disulfide, disulfide/native thiol, disulfide/total thiol, native thiol/total thiol and IMA. The values for sensitivity and specificity are shown in Table 5.

## Discussion

Evidence of OS due to obesity in adults and more recently, evidence in children, has appeared over the last few years (43). Obesity creates pro-oxidant conditions that promote the development of comorbid diseases. Energy imbalance leads to storage of excess energy in adipocytes, resulting in both hypertrophy and hyperplasia. These processes are related to abnormalities of adipocyte function, especially mitochondrial stress and impaired endoplasmic reticulum function (44,45). OS can also be induced by adipocyte-associated inflammatory macrophages (46). There is a close association between obesity, chronic low-level inflammation and OS. In addition, the dysfunction of adipocytokines secreted by adipose tissue and induced by OS, act synergistically in metabolic abnormalities associated with obesity. Evaluation of oxidative status has been reported to aid in identification of patients with high risk of complications (43).

Thiol-disulfide balance has vital importance and the new method developed by Erel and Neselioglu (41) is capable of measuring both separate variables and providing an overall picture of thiol-disulfide balance while allowing both individual and integral evaluations. Until now, many studies have evaluated oxidant-antioxidant status and reported various results for obese children. However, to the best of our knowledge, no previous study has reported thiol/disulfide homeostasis in MUO and MHO children. Elmas et al (47) first evaluated thiol/disulfide homeostasis in obese children, reporting that antioxidant parameter levels were low in obese patients, while pro-oxidant parameters were elevated. In contrast to Elmas et al (47) we measured thiol/disulfide homeostasis in MHO and MUO children for the first time in order to investigate differences in oxidant/antioxidant balance between these two different groups of obese children.

In our study, the concentrations of native thiol and total thiol and native thiol/total thiol ratio were lower while disulfide concentration and disulfide/native thiol and disulfide/total thiol ratios were higher in obese children than the healthy control group. This suggests a shift in thiol/disulfide homeostasis towards disulfide production. Oxidant parameters were high and anti-oxidant parameters were low in obese children. When obese children were divided into MUO and MHO groups, parameters of OS in the MUO group were higher compared to the healthy control group. Interestingly, there was also no difference between the MUO and MHO groups. Development of chronic inflammation due to OS indicated by metabolically impaired parameters in obese children has been shown to lead to disruption of thiol redox homeostasis. Our data suggest that the increased pro-oxidant status in obese children worsens with metabolic abnormalities.

Many previous studies have found increased pro-oxidant status in obese individuals, similar to our study. Vehapoğlu et al (48) found that antioxidant capacity was significantly lower in prepubertal obese children. Karamouzis et al (49) demonstrated that loss of normal oxidant-antioxidant homeostatic balance led to increased OS with decreased antioxidant capacity in obese prepubertal and adolescent girls. Paltoglou et al (50) found that childhood obesity was associated with aseptic inflammation and OS. In another study investigating the changes in the oxidant/antioxidant homeostasis in obese children with and without metabolic syndrome, a significant impairment in the oxidant/antioxidant balance was reported in those with metabolic syndrome (51). Another study also showed that children were more susceptible to OS than adults and the authors suggested that this was probably due to the incomplete development of the antioxidant system (52). The results of our study were consistent with those reported in studies on obesity and excessive OS. We suggest that thiol/disulfide homeostasis in MUO children may be a reliable indicator of oxidant-antioxidant status.

An increased degree of obesity has been shown to predict increased metabolic risk in obese children and adolescents (53). When compared to their moderately obese peers, the severely obese are at greater risk for adult obesity, early atherosclerosis, hypertension, type 2 diabetes, metabolic syndrome, fatty liver disease and premature death (54). In our study, there was no differences in thiol/disulfide homeostasis parameters and IMA between the MUO and MHO children with >3 BMI-SDS. This result may be significant for metabolic risk that may develop in metabolically healthy but seriously obese children and adolescents. It would be necessary to undertake longer term studies of the seriously obese but metabolically healthy children in order to determine if they will progress to the unhealthy group and what the natural history of this progression in terms of thiol/disulfide parameters might be.

Many previous studies assessed serum IMA levels in adult obese patients and found correlations between some anthropometric and laboratory measurements. Piva et al (55) and Kazanis et al (56) reported that serum IMA concentration was significantly elevated in obese adults and overweight/obese postmenopausal women and this was associated with OS. In addition, both studies found an association between serum IMA concentration and BMI. Baysal et al (26) investigated studied serum IMA in obese children for the first time and found higher levels in children with metabolic syndrome. Similarly, in our study, serum IMA concentration was higher in the obese group than the control group although we could not detect a difference between the MUO and MHO groups. We found positive correlations between serum IMA concentration and BMI, fasting blood glucose, insulin, and HOMA-IR levels.

The IMA variable has the highest AUC value of the parameters examined for predicting MUO and at a cut-off value of 0.665 ABSU the sensitivity and specificity were 74% and 66%, respectively. Both native thiol concentration and total thiol values had high specificity at the identified cut-offs (439.2 µmol/L and 477.5 µmol/L, respectively) suggesting that they are likely to differentiate between MUO and healthy individuals. The sensitivity and specificity of the disulfide parameter, relative to the cut-off value (23.18 µmol/L), were 79% and 43%, respectively. Here, the sensitivity was good suggesting that it may be useful in distinguishing between high values and possibly unhealthy patients.

### Study Limitations

An important limitation is that this study is cross-sectional. Furthermore, thiol/disulphide parameters were not compared with other enzymatic and non-enzymatic OS parameters.

## Conclusion

In this study, we showed that thiol/disulfide homeostasis, one of the important parameters of OS, is impaired in MUO children. Our data prove that the increased oxidant status in obese children is related to the metabolic abnormality. Measurable increases in OS may be the basis of obesity-related comorbidities. Reducing chronic inflammation and OS levels in childhood can prevent subsequent metabolic disorder as well as increased cardiovascular morbidity and mortality in adulthood. Our study provides an idea about these issues; however, future in-depth studies are warranted.

## Figures and Tables

**Table 1:**
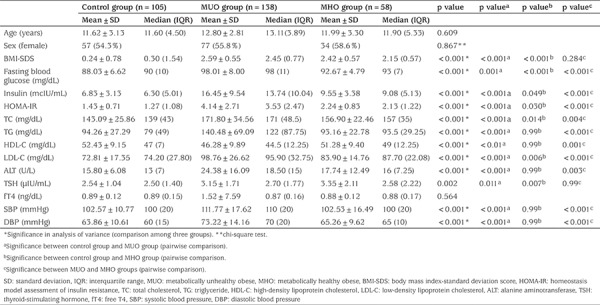
Demographic, clinical and laboratory characteristics of participants

**Table 2:**
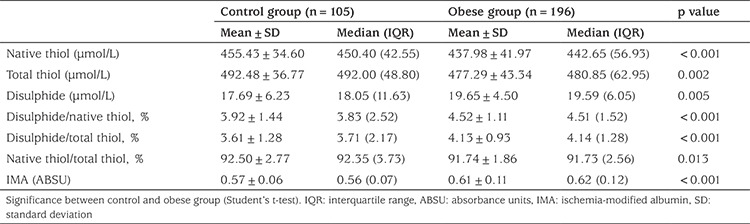
Thiol/disulfide homeostasis parameters and comparison of ischemia-modified albumin between the control group and all obese patients

**Table 3:**
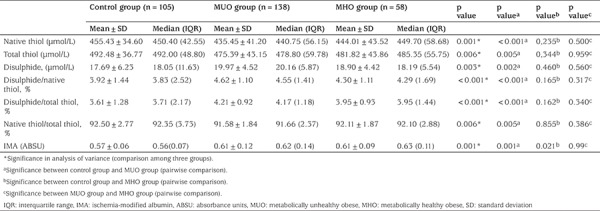
Comparison of dynamic thiol/disulfide homeostasis and ischemia-modified albumin between the control, metabolically unhealthy obese and metabolically healthy obese groups

**Table 4:**
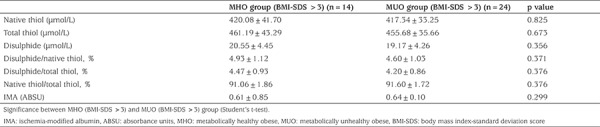
Comparison of dynamic thiol/disulfide homeostasis and ischemia-modified albumin between subgroups with body mass index-standard deviation score >3 from both the metabolically healthy obese and metabolically unhealthy obese groups

**Table 5:**
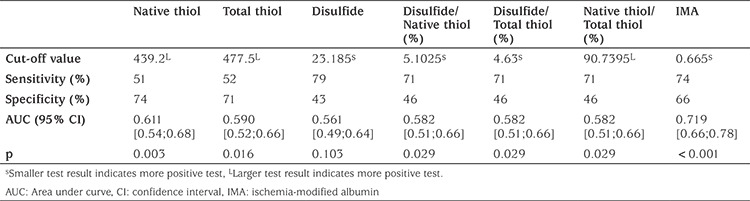
The cut-off values of parameters in predicting metabolically unhealthy obese group
